# Interactive Effects of Genotype and Molybdenum Supply on Yield and Overall Fruit Quality of Tomato

**DOI:** 10.3389/fpls.2018.01922

**Published:** 2019-01-04

**Authors:** Leo Sabatino, Fabio D'Anna, Giovanni Iapichino, Alessandra Moncada, Eleonora D'Anna, Claudio De Pasquale

**Affiliations:** Dipartimento Scienze Agrarie, Alimentari e Forestali, Università di Palermo, Palermo, Italy

**Keywords:** trace element, *Solanum lycopersicum* L., crop performance, nutraceutical compounds, soilless system

## Abstract

Molybdenum (Mo) is an essential trace element for plant growth, development, and production. However, there is little known about the function and effects of molybdenum in tomato plants. The present study assessed the influences of different Mo concentrations on four tomato F_1_ hybrids (“Bybal” F_1_, “Tyty” F_1_, “Paride” F_1_, and “Ornela” F_1_) grown using a soilless system with different Mo levels [0.0, 0.5 (standard NS), 2.0, and 4.0 μmol L^−1^, respectively]. The crop yield, plant vigor, fruit skin color, TA, fruit water content as well as the accumulation of SSC, and some antioxidant compounds such as lycopene, polyphenols and ascorbic acid were evaluated. The minerals concentration, including nitrogen (N), Mo, iron (Fe), and copper (Cu), were measured in tomato fruits. Results revealed that tomato plants grown with 2.0 μmol Mo L^−1^ compared to plants grown with 0.5 μmol Mo L^−1^ incurred a significant increase of total yield by 21.7%, marketable yield by 9.1%, aboveground biomass by 16.7%, plant height at 50 DAT by 6.5%, polyphenol content by 3.5%, ascorbic acid by 1.0%, SSC by 3.5%, N fruit content by 24.8%, Mo fruit content by 20.0%, and Fe fruit content by 60.5%. However, the Mo concentration did not significantly influence the average fruit weight, b^*^ fruit skin color coordinate and TA. Furthermore, tomato fruits from plants grown with 2.0 μmol Mo L^−1^ showed a lower Cu fruit content (16.1%) than fruits from plants grown with 0.5 μmol Mo L^−1^ (standard NS). Consequently, our study highlights the different behavior of the tomato genotypes tested when subjected to different levels of Mo concentration in the nutrient solution. Nevertheless, taking all in consideration our results clearly suggest that a Mo fertilization of 2.0 μmol Mo L^−1^ effectively enhance crop performance and overall fruit quality of tomato.

## Introduction

For human body, molybdenum (Mo) belongs to the category of trace elements, which are needed in very small quantities (generally <100 mg day^−1^), as opposed to elements considered macronutrients, such as nitrogen, phosphorus, sodium, calcium, magnesium, potassium, chlorine, etc., which are required in larger quantities (Fraga, 2005; Ierna et al., 2012). Tsongas et al. (1980) calculated a daily optimal intake of 120–240 μg Mo day^−1^, depending on age, sex, and income.

The severity of the pathology related with simple sulfite oxidase deficiency, first described by Mudd et al. (1967), and the subsequent characterization of the enzyme as a molybdenum-containing protein by Cohen et al. (1971), confirmed the essentiality of Mo for normal human development. The marked neurological lesions observed since then in several patients exhibiting combined deficiency of all molybdenum-containing enzymes (Johnson et al., 1974) reinforce the importance of the metal in human health (Rajagopalan, 1988). One of the findings made by some authors was a relationship between Mo deficiency and esophageal cancer (Burrell et al., 1966). Furthermore, Gunnison (1981) reported an increased incidence of mammary adenocarcinoma in sulfite-oxidase-deficient (tungsten-treated) rats.

Mo, such as other trace elements, is an essential component of biological structures, but simultaneously it can be toxic at concentrations beyond those necessary for their biological functions. Luk et al. (2003) found that to deal with this essentiality/toxicity duality, biological systems have developed the ability to recognize a metal, and deliver it to the target without allowing the metal to participate in toxic reactions. As other metals, Mo itself is inactive in biological systems until it is part of an organic pterin complex called pterin-based molybdenum cofactor (Moco) (Schwarz et al., 2009). Mo is found in nearly all foods in trace amounts as soluble molybdates. Generally, foods rich in Mo are legumes, cereals, cereal products, and nuts (Pennington and Jones, 1987). Cereals and cereal products, such as bread or pasta are the major food contributors to dietary molybdenum intake of adults in Western countries, followed by dairy products and leafy vegetables (Pennington and Jones, 1987; Rose et al., 2010), whereas, fruits, stem, and root are among the poorest (Rajagopalan, 1988).

The importance of molybdenum for plants is well known and documented (Mulder, 1954; Mendel and Schwarz, 1999; Zimmer and Mendel, 1999) and was first reported by Arnon and Stout (1939). The phenotypic alteration of Mo-deficient plants is characterized by altered morphology of leaves, impaired flower formation, poor fruit quality, and an overall stunting in plant growth and development. Longbottom et al. (2010) reported that Mo foliar sprays on Merlot grapevines is an effective mean to increase yield and berry size. In addition, Eshghi et al. (2010) revealed that Mo increased pollen germination rate of strawberry. However, to our knowledge, little or no research has been conducted on the role of Mo on tomato production and overall fruit quality.

Mo influences the biochemical processes and chemical composition of plants (Kostova and Mehandjiev, 2013). As in other organisms, plants utilize Mo in selected enzymes such as nitrate reductase, xanthine dehydrogenase, aldehyde oxidase, sulphite oxidase, and the mitochondrial amidoxime-reducing component (mARC1 and mARC2; Schwarz, 2016). These enzymes carry out redox reactions, in particular, in processes involving nitrogen metabolism (Kaiser et al., 2005), phytohormone biosynthesis, purine metabolism, sulfite detoxification, and the reduction of a broad range of N-hydroxylated compounds (Hille et al., 2011). Increasing demand for high-quality fruits with good color and rich in compounds important for human health has led to a proliferation in research on fresh fruit quality, including physicochemical characteristics. In this respect, yield, apparent fruit quality traits, and chemical composition of the fruits from Mo enriched plants should remain equal or improved with respect to the Mo no-enriched plants.

Tomato (*Solanum lycopersicum* L.) is the world's most important vegetable crop (FAO STAT, 2016; http://faostat3.fao.org/browse/Q/QC/E) with a worldwide production of over 161 million tons worth over €808 000 million in 2016 (Davino et al., 2017). Although, nowadays, there are emerging class of specialty crops such as microgreens, tomato is not affected by any reduction in consumption. Kostova (2010) reported that a high concentration of Mo has positive influence upon the content of antioxidants in the fruit of tomatoes. However, to the best of our knowledge, the literature lacks information on the interaction between tomato genotypes and Mo concentration in the nutrient solution. Therefore, starting from the above-mentioned considerations, the aims of the present study are as follows: (i) to investigate on the impact of different levels of Mo concentration in the nutrient solution on the Mo fruit content of different tomato genotypes and (ii) to assess the yield and fruit nutritional quality of different tomato genotypes that are grown using nutrient solutions with different Mo concentrations.

## Materials and Methods

### Plant Material and Experimental Conditions

The research was conducted in an experimental field of the Department of Agricultural, Food, and Forest Sciences of Palermo (SAAF), at Marsala, Trapani Province (longitude 12°26'E, latitude 37°47'N, altitude 37 m) in the North-western coast of Sicily (Italy). A 27 × 50 m, north-south oriented, multi-span greenhouse covered with polyvinyl chloride was used for the experiment. The high-tech greenhouse was equipped with a fan-and-pad evaporative cooling, high-pressure fogging and over-head air heating systems.

On 18th February 2016, seedlings of four tomato hybrids [“Bybal” F_1_ (Syngenta Seed, Basel, Switzerland) belonging to the round tomato group, “TyTy” F_1_ (Syngenta Seed, Basel, Switzerland) belonging to the cherry tomato group, “Paride” F_1_ (MedHermes, Ragusa, Italy), and “Ornela” F_1_ (Vilmorin Italia, Bologna, Italy) belonging to the ellipsoid tomato group], raised in peat, were transferred to a perlite grow-bags [Agripan perlite (Perlite Italiana, Milan, Italy)] at the stage of four to five true leaves. Each bag was 1.0 m in length, 0.25 m in width, and 33 L in volume and accommodated four tomato plants. The plant density was 3.3 plants m^−2^.

As a second treatment factor, Mo in the nutrient solution (NS) was adjusted to a null, a low (standard) and two high concentrations corresponding to 0.0, 0.5, 2.0, and 4.0 μM Mo, respectively, from the beginning to the end of the experiment. The different Mo levels were attained by adding appropriate amounts of ammonium molybdate tetrahydrate to the nutrient solution. The concentrations of all other nutrients in the solution initially introduced into the system were identical for all NS treatments and the composition was as follows: 1.2 mM NH${}_{4}^{+}$, 9.5 mM K^+^, 5.4 mM Ca^2+^, 2.4 mM Mg ^2+^, 16.0 mM NO${}_{3}^{-}$, 4.4 mM SO${}_{4}^{2-}$, 1.5 mM H_2_PO_4_, 15.0 μM Fe, 10.0 μM Mn, 5.0 μM Zn, 30 μM B, and 0.75 μM Cu (Sonneveld and Voogt, 2009). In addition, the irrigation water contained 9.5 mM of Na and 9.0 mM of Cl. The electrical conductivity (EC) and pH in the above NS were 3.60 mS cm^−1^ and 5.6, respectively. The pH in the NS was adjusted to 5.6 to 5.7 daily by adding appropriate amount of HNO_3_. Plants were fed by complete NS given daily via drip irrigation system. In-line emitters with discharge rate of 2.0 L h^−1^ at 0.25 m spacing on lateral were used. The amount of water was estimated according to the solar radiation of the previous day (Boztok et al., 1984; Gül and Sevgican, 1992). A leaching fraction of 40% was adopted. The drainage was collected in a reservoir tank, however, it was not reutilized (open cycle management).

The four Mo treatments were combined with the four tomato genotype treatments in a two-factorial experimental design rendering 16 treatments. Each treatment was replicated four times and contained four plants. Thus, the total number of tomato plants was 256. Fruit setting was facilitated by vibration of the trusses at approximately midday two times a week. Climate conditions inside the greenhouse were adjusted via computer controller. In order to avoid limitations in fruit setting resulting from insufficient pollen production or pollen tube growth, the inside air temperature was set to 16 ± 1°C during the night and 24 ± 1°C during the day. Relative humidity was kept between 60 and 70% during the growing season of tomato. The cumulated greenhouse global radiation was 1615.1 MJ m^−2^.

### Plant Vigor, Flower Emission, Yield, and Apparent Fruit Quality Evaluation

Plant vigor was assessed by plant height at 50 days after transplanting (DAT) and aboveground biomass produced at the end of fruit harvest [including total yield and vegetative part produced (weight of the plant at the end of harvests plus vegetative part removed by pruning)]. First truss emission (expressed as DAT) wasalso collected.

Immediately after harvesting fruits were weighed. Total yield (kg plant^−1^) and marketable yield (kg plant^−1^) were estimated. Average fruit weight (g) was also calculated.

Immediately after harvesting, fruit color (L^*^, a^*^, and b^*^ parameters -CIELab) was measured on four replications of five fruits per treatment. The records were taken on two opposite point of tomato fruit skin (equatorial zone) by a colorimeter (Chroma-meter CR-400, Minolta Corporation, Ltd., Osaka, Japan). The colorimeter was calibrated with a white standard calibration plate (Y = 93.9, x = 0.3134, y = 0.3208) before use. L^*^ corresponds to a dark/light scale (0 = black, 100 = white) and represents the relative lightness of colors, being low for dark colors, and high for light colors (McGuire, 1992; Lancaster et al., 1997).

### Proximate Composition and Fruit Mineral Content

Sampling for the fruit quality analysis was conducted as described by Sabatino et al. (2016, 2018) for eggplant. Thus, 3–5 commercially mature fruits for each replication from the second and third harvest were used; only healthy fruits were chosen. Care was taken to ensure that each sample contained the same percentage weight of apical, middle, and distal parts of the fruits. Qualitative fruit characteristic analyses were conducted on fruits harvested from labeled fruits (the flowers were labeled at the fruit set stage) and all fruits were harvested after 35 days from labeling (fruit commercial maturity stage).

Samples of the fruit pulp were squeezed by hand with a garlic squeezer. The juice was filtered and soluble solids content (SSC) was measured using a digital refractometer (MTD-045 nD, Three-In-One Enterprises Co. Ltd. Taiwan).

Titratable acidity (TA) was determined using 10 g aliquots of tomato fruits poured in 50 mL of distilled water and titrated with 0.1 N NaOH to an end-point of pH 8.1. TA was expressed as percentage of citric acid and was calculated using the method reported by Han et al. (2004). The SSC/TA ratio was also calculated.

Fruit water content was determined in samples dried at 80°C for the first two days and subsequently dried at 105°C until constant weight using a thermo-ventilated oven (Memmert, Serie standard, Venice, Italy). Water content (%) was calculated from the difference in the masses before and after drying.

Ascorbic acid content was measured from tomato samples by reflectometer Merck RQflex^*^ 10 meter using Reflectoquant Ascorbic Acid Test Strips. One gram of fruit juice was dissolved in distilled water, maked up to 10 mL, and mixed; then dipped appropriate test strip into the sample and inserted it into the meter. Results were expressed as mg of ascorbic acid per 100 g fresh weigh.

Total phenolic content was measured by using 2 g of each sample which was weighed out and extracted with 50 ml of methanol. The extraction was conducted under stirring for 60 min at 60°C. The mixture was filtered through filter paper (Whatman No. 3), filled in a 50 ml volumetric flask and allowed to set in the dark until analysis. Total phenolic content was determined according to the Folin-Ciocalteu method (Slinkard and Singleton, 1997) with slight modifications. The standard or sample extract (100 μL; triplicate) was mixed with 0.4 mL Folin-Ciocalteu reagent. After 3 min reaction 0.8 mL of 10 % Na_2_CO_3_ was added. The tubes were allowed to stand for 30 min at room temperature, and the absorption was measured at 765 nm using a spectrophotometer (CELL, model CE 1020, Cambridge, UK). Gallic acid was used as calibration standard, and the results were calculated as gallic acid equivalent (GAE; mg 100 g^−1^ dry weight basis).

Lycopene content was determined as described by Sadler et al. (1990). Briefly, 5 g of homogenized sample was extracted adding 50 ml of a mixture of hexane/acetone/ethanol (2:1:1, v/v/v) for 30 min. The total lycopene content expressed in mg 100 g^−1^ fresh weight was obtained by measuring the absorbance of the lycopene hexane fraction at 472 nm. Pure lycopene (Sigma, St. Louis, MO) was used for the preparation of calibration curves.

Nitrogen (N) content was obtained from the Kjeldahl method. In particular, a sample rate was subjected to acid-catalyzed mineralization to turn the organic nitrogen into ammoniacal nitrogen. The ammoniacal nitrogen was then distilled in an alkaline pH. The ammonia formed during this distillation was collected in a boric acid solution and determined through titrimetric dosage.

The Mo was determined by ICP-MS instrument (Plasma Quant MS Elite, Jena, Germany), roughing pump, re-circulator, data acquisition and analysis software, equipped with a low liquid uptake nebulizer, a free-running radio frequency (RF) plasma generator, automated X, Y, Z torch positioning, and a four-stage vacuum system. A MARS6 (CEM, USA) high-throughput closed microwave digestion workstation was used for dissolving metal and preparing reference solution.

Iron (Fe) and copper (Cu) were determined using atomic absorption spectroscopy (SavantAA, ERRECI, Milan, Italy) following wet mineralization as reported by Morand and Gullo (1970).

### Statistical Analysis

The data were subjected to two-factorial analysis of variance (genotype × Mo concentration) using the SPSS software package version 14.0 (StatSoft, Inc., Chicago, USA). For data expressed in percentage, the arcsin transformation before ANOVA analysis [Ø = arcsin(p/100)^1/2^] was applied. When the genotype and/or the Mo supply level were significant, the means were separated using Tukey HSD test (*P* < 0.05). The same test was used to separate the 16 means from all experimental treatments when the interaction for a particular measured characteristic was significant.

## Results

### Crop Performance

As regard total yield, a significant interaction was found between genotype and Mo concentration (Table 1). The highest total yields were identified in “Bybal” F_1_ plants grown with 2.0 μmol Mo L^−1^ and in “Bybal” F_1_ plants grown with 2.0 μmol Mo L^−1^. The lowest total yield was observed in ‘Tyty' F_1_ tomato genotype grown using a NS with 0.0 μmol Mo L^−1^.

**Table 1:** Interactive effects of genotype (“Bybal” F_1_, “TyTy” F_1_, “Paride” F_1_, and “Ornela” F_1_) and molybdenum supply level [0.0, 0.5 (standard NS), 2.0, and 4.0 μM L^−1^] on total yield, marketable yield, aboveground biomass, a^*^ color coordinate, ascorbic acid, SSC, TA, SSC/TA, N fruit concentration, and Mo fruit concentration.

	**Total yield (kg plant^**−1**^)**	**Marketable yield (kg plant^**−1**^)**	**Aboveground biomass (kg)**	a^*^	**Ascorbic acid (mg 100 g^**−1**^ of dw)**	**SSC (^**°**^Brix)**	**TA (% citric acid)**	**SSC/TA**	**N (g 100 g^**−1**^ of dw)**	**Mo (mg kg^**−1**^ of dw)**
“Bybal” F_1_ × 0.0 μmol Mo L^−1^	2.8d	2.6c	5.3c	11.8c	1978.0b	6.4g	0.5c	14.3e	0.64h	0.02e
“Bybal” F_1_ × 0.5 μmol Mo L^−1^	3.4c	3.3b	6.1b	11.9c	1978.0b	7.2f	0.5c	16.0cd	1.05e	0.06c
“Bybal” F_1_ × 2.0 μmol Mo L^−1^	4.6a	4.0a	7.5a	14.0b	1677.0d	8.0e	0.5c	17.2b	1.3c	0.07c
“Bybal” F_1_ × 4.0 μmol Mo L^−1^	3.8b	3.5b	5.4c	15.0ab	1730.8cd	5.3h	0.5bc	11.2g	0.78g	0.07c
“Tyty” F_1_ × 0.0 μmol Mo L^−1^	1.1i	1.0g	2.4g	13.7b	1816.2c	8.6d	0.6a	15.2d	0.55i	0.02e
“Tyty” F_1_ × 0.5 μmol Mo L^−1^	1.3i	1.2f	2.8f	13.8b	1816.2c	9.6b	0.6a	17.0bc	0.94f	0.03d
“Tyty” F_1_ × 2.0 μmol Mo L^−1^	1.4i	1.1fg	2.9f	14.1b	1530.8e	8.9c	0.6a	15.9cd	1.18d	0.03d
“Tyty” F_1_ × 4.0 μmol Mo L^−1^	1.4i	1.2f	2.9f	11.8c	1575.8d	8.9c	0.5a	16.6c	0.65h	0.06c
“Paride” F_1_ × 0.0 μmol Mo L^−1^	2.2f	2.0d	3.8de	5.5d	1940.3b	7.0f	0.5c	15.3d	0.63h	0.03d
“Paride” F_1_ × 0.5 μmol Mo L^−1^	2.6e	2.5c	4.3d	5.6d	1940.3b	7.8e	0.5c	17.1b	1.21d	0.06c
“Paride” F_1_ × 2.0 μmol Mo L^−1^	3.5c	3.2b	5.4c	5.7d	1621.4de	9.3b	0.5b	18.5a	1.52a	0.07c
“Paride” F_1_ × 4.0 μmol Mo L^−1^	3.4c	3.1b	3.5e	5.4d	1617.2d	6.3g	0.5b	12.3f	0.75g	0.13a
“Ornela” F_1_ × 0.0 μmol Mo L^−1^	1.5h	1.3f	3.3f	15.9a	2023.4b	8.7c	0.6a	15.8cd	0.66h	0.04d
“Ornela” F_1_ × 0.5 μmol Mo L^−1^	1.8g	1.7e	3.7e	16.1a	2023.4b	9.7a	0.6a	17.7b	1.14d	0.04d
“Ornela” F_1_ × 2.0 μmol Mo L^−1^	1.8g	1.6e	3.9e	16.9a	3008.8a	9.3b	0.6a	16.9c	1.42b	0.05d
“Ornela” F_1_ × 4.0 μmol Mo L^−1^	1.9fg	1.7e	3.9e	16.9a	1805.3c	8.5d	0.6a	15.1d	0.76	0.11b
**SIGNIFICANCE**										
Genotype	^***^	^***^	^***^	^***^	^***^	^***^	^***^	^***^	^***^	^***^
Mo concentration	^***^	^***^	^***^	^**^	^***^	^***^	NS	^***^	^***^	^***^
Interaction	^***^	^***^	^***^	^***^	^***^	^***^	^*^	^***^	^**^	^***^

Data within a column followed by the same letter are not significantly different at p ≤ 0.05 according to Tukey HSD Test. The significance is designated by asterisks as follows:

* , statistically significant differences at p-value below 0.05;

** , statistically significant differences at p-value below 0.01;

*** *, statistically significant differences at p-value below 0.001; NS = not significant*.

The data collected on marketable yield supported the trend established for total yield (Table 1).

Regardless of the genotype, Mo concentration did not significantly affect average fruit weight (Table 2). Conversely, the genotype significantly influenced the average fruit weight which was highest in fruits from “Bybal” F_1_ plants and lowest in “Ornela” F_1_ plants. No significant interaction was found between genotype and Mo concentration in terms of average fruit weight.

**Table 2:** Analysis of variance and mean comparisons for average fruit weight, plant height 50 DAT, first truss emission, L*, b*, lycopene, polyphenol content, fruit water content, Fe fruit concentration and Cu fruit concentration of four tomato genotypes treated with different molybdenum supply levels in the nutrient solution introduced to the soilless system.

**Treatments**	**Average fruit weight (g)**	**Plant height 50 DAT (cm)**	**First truss emission (DAT)**	L*	b*	**Lycopene (mg 100 g^**−1**^ of dw)**	**Polyphenol content (mg gallic acid eq. 100 g^**- 1**^ of dw)**	**Fruit water content (%)**	**Fe (mg kg^**−1**^ of dw)**	**Cu (mg kg^**−1**^ of dw)**
**GENOTYPE**										
“Bybal” F_1_	179.7a	85.1a	23.3c	48.5a	23.0a	878.9ab	17.5b	94.1b	18.41a	3.81a
“Tyty” F1	32.8c	77.0 b	25.6a	50.3a	19.1bc	909.1a	17.5b	93.5c	16.12a	3.61b
“Paride” F1	117.5b	83.4a	25.4a	39.1b	18.7c	920.2a	18.0a	94.5a	18.34a	3.80a
“Ornela” F1	20.0d	84.8a	24.4b	49.3a	20.1b	837.8b	17.3b	92.7d	17.59a	3.87a
**Mo CONCENTRATION (μmol/L)**										
0.0	87.3a	71.9d	23.4b	48.1a	20a	886.7a	16.9c	94.2a	13.67c	4.37a
0.5 (standard NS)	87.3a	87.8b	23.4b	48.1a	20a	925.3a	17.1c	94.2a	15.23bc	4.26a
2.0	87.7a	93.5a	25.9a	47.8a	20a	914.0a	17.7b	93.2b	24.44a	3.67b
4.0	87.7a	82.3c	25.9a	43.0b	21a	820.1b	18.4a	93.0c	17.11b	2.78c
**SIGNIFICANCE**										
Genotype	^***^	^***^	^***^	^***^	^***^	^***^	^***^	^***^	NS	^***^
Mo concentration	NS	^***^	^***^	^**^	NS	^***^	^***^	^***^	^***^	^***^
Interaction	NS	NS	NS	NS	NS	NS	NS	NS	NS	NS

Data within a column followed by the same letter are not significantly different at p ≤ 0.05 according to Tukey HSD Test. The significance is designated by asterisks as follows:

** , statistically significant differences at p-value below 0.01;

*** *, statistically significant differences at p-value below 0.001; NS = not significant*.

ANOVA for aboveground biomass showed a significant effect of the interaction genotype × Mo concentration (Table 1). “Bybal” F_1_ plants grown using a NS with a Mo concentration of 2.0 μmol L^−1^ had the highest aboveground biomass value, followed by plants of the same cultivar grown at a Mo concentration of 0.5 μmol L^−1^. “Tyty” F_1_ plants grown using a NS with a Mo concentration of 0.0 μmol L^−1^ had the lowest aboveground biomass.

Regardless of the Mo concentration, “Bybal” F_1_, “Paride” F_1_, and “Ornela” F_1_ showed the highest values in terms of plant height at 50 DAT, whereas, “Tyty” F_1_ showed the lowest one (Table 2). Irrespective of the genotype, tomato plants grown using a NS with a Mo concentration of 2.0 μmol L^−1^ displayed the highest plant height. Plants grown with 0.0 μmol Mo L^−1^ showed the lowest value in terms of plant height. ANOVA for plant height at 50 DAT did not show a significant interaction genotype × Mo concentration.

Ignoring of the Mo concentration, “Bybal” F_1_ plants gave the shortest time of first truss emission, whereas, “Tyty” F_1_ and “Paride” F_1_ revealed the longest first truss emission time (Table 2). Irrespective of the genotype, tomato plants grown using a NS with a Mo concentration of 0.0 and 0.5 μmol L^−1^ displayed a shorter time in terms of first truss emission compared to tomato plants grown with 2.0 and 4.0 μmol Mo L^−1^. No significant interaction genotype × Mo concentration was found.

### Intrinsic and Extrinsic Tomato Fruit Quality

Regardless of the Mo concentration (Table 2), “Bybal” F_1_, “Tyty” F_1_, and “Ornela” F_1_ displayed the highest L^*^ color coordinate, whereas, “Paride” F_1_ showed the lowest one. Irrespective of the genotype, tomato plants grown using a NS with a Mo concentration of 0.0, 0.5, and 2.0 μmol L^−1^ revealed the highest L^*^ color coordinate. Whereas, tomato plants grown using NS with a Mo concentration of 4.0 μmol L^−1^ showed the lowest L^*^ color coordinate. ANOVA for L^*^ color coordinate did not show a significant interaction genotype × Mo concentration.

ANOVA for a^*^ color coordinate showed a significant effect of the interaction genotype × Mo concentration (Table 1). “Ornela” F_1_ plants grown using a NS with a Mo concentration of 0.0, 0.5, 2.0, and 4.0 μmol L^−1^ had the highest a^*^ fruit color coordinate value (Table 1), followed by “Bybal” F_1_ grown with 4.0 μmol Mo L^−1^ which in turn showed values 7.1% higher than plants of the same cultivar grown at a Mo concentration of 2.0 μmol Mo L^−1^. “Paride” F_1_ plants grown using a NS with a Mo concentration of 4.0 μmol L^−1^ had the lowest a^*^ fruit color coordinate.

Regardless of the genotype, Mo concentration did not significantly affect b^*^ fruit color coordinate (Table 2). On the contrary, the genotype significantly influenced the aforementioned parameter. The highest b^*^ fruit color coordinate value was recorded from “Bybal” F_1_, followed by “Ornela” F_1_. The lowest b^*^ fruit color coordinate was recorded from “Paride” F_1_. No significant interaction was found between genotype and Mo concentration in terms of b^*^ fruit color coordinate.

Disregarding of the Mo concentration (Table 2), fruits from “Tyty” F_1_ and “Paride” F_1_ showed the highest lycopene content, while, fruits from “Ornela” F_1_ revealed the lowest values. However, fruits from “Bybal” F_1_ did not show significant difference neither from fruits from “Tyty” F_1_ and “Paride” F_1_ nor from those from “Ornela” F_1_ in terms of lycopene content. Without regard of the genotype, fruits from tomato plants grown using a NS with 0.0, 0.5, and 2.0 μmol Mo L^−1^ gave the highest lycopene content, whereas, those from tomato plants grown with 4.0 μmol Mo L^−1^ gave the lowest values (Table 2). No significant interaction was found between genotype and Mo concentration in terms of fruit lycopene content.

Ignoring of the Mo concentration, “Paride” F_1_, displayed the highest polyphenol content, while, “Bybal” F_1_, “Tyty” F_1_, and “Ornela” F_1_ showed the lowest ones (Table 2). Irrespective of the genotype, at 4.0 μmol Mo L^−1^ polyphenol content was 4.0% significantly higher than that recorded in fruits from plants grown with a Mo concentration of 2.0 μmol L^−1^. The lowest polyphenol content was found in fruits from tomato plants grown using a NS with 0.0 and 0.5 μmol Mo L^−1^. ANOVA for polyphenol content did not show a significant interaction genotype × Mo concentration (Table 2).

With respect to ascorbic acid, ANOVA showed a significant effect of the interaction genotype × Mo concentration (Table 1). The combination “Ornela” F_1_ × 2.0 μmol Mo L^−1^ had the highest ascorbic acid value, followed by “Ornela” F_1_, “Paride” F_1_, and “Bybal” F_1_ grown with 0.0 and 0.5 μmol Mo L^−1^ which in turn showed a higher ascorbic acid value than “Tyty” F_1_ grown with 0.0 and 0.5 μmol Mo L^−1^ and “Ornela” F_1_ grown using a NS with 4.0 μmol Mo L^−1^. The combination “Tyty” F_1_ × 2.0 μmol L^−1^ produced the lowest ascorbic acid level.

As regarding the fruit water content, ignoring of the Mo concentration the highest value was recorded from “Paride” F_1_, followed by “Bybal” F_1_ (Table 2). The lowest fruit water content was recorded from “Ornela” F_1_. Disregarding of the genotype, tomato plants grown at 0.0 and at 0.5 μmol Mo L^−1^ displayed the highest fruit water content value which in turn was significantly higher value than thet recorded in plants grown at a Mo concentration of 2.0 μmol L^−1^. Plants grown with 4.0 μmol Mo L^−1^ showed the lowest fruit water content. No significant interaction was found between genotype and Mo concentration in terms of fruit water content.

ANOVA for SSC revealed a significant effect of the interaction genotype × Mo concentration (Table 1). “Ornela” F_1_ grown at a Mo concentration of 0.5 μmol L^−1^ had the highest SSC value, which was 4.3, 4.3, and 1.0%, respectively higher than that recorded in “Ornela” F_1_ grown with 2.0 μmol Mo L^−1^, in “Paride” F_1_ grown with 2.0 μmol Mo L^−1^ and in “Tyty” F_1_ grown with 0.5 μmol Mo L^−1^. “Bybal” F_1_ grown using a NS with a Mo concentration of 4.0 μmol L^−1^ showed the lowest SSC value.

ANOVA for TA showed a significant effect of the interaction genotype × Mo concentration (Table 1). The highest TA was found in fruits from “Ornela” F_1_ and “Tyty” F_1_ which in turn was significantly higher than in fruits from “Paride” F_1_ grown using a NS with a Mo concentration of 2.0 and 4.0 μmol L^−1^. The lowest values of TA were recorded in fruits from “Bybal” F_1_ grown with 0.0, 0.5 (standard NS) and 2.0 μmol Mo L^−1^ and in fruits from “Paride” F_1_ grown with 0.0, 0.5 μmol Mo L^−1^.

In respect to the SSC/TA ratio, a significant interaction was found between genotype and Mo concentration (Table 1). The highest SSC/TA ratio was detected in fruits from “Paride” F_1_ grown using a NS with a Mo concentration of 2.0 μmol L^−1^, whereas, the lowest one was identified in fruits from “Bybal” F_1_ grown with 4.0 μmol Mo L^−1^.

ANOVA for N fruit concentration showed a significant interaction genotype × Mo concentration (Table 1). Fruits from “Paride” F_1_ plants grown using a NS with a Mo concentration of 2.0 μmol L^−1^ showed the highest N content followed by those from “Ornela” F_1_ grown at 2.0 μmol Mo L^−1^ which in turn had a N content 8.4% higher than fruits from “Bybal” F_1_ grown with 2.0 μmol Mo L^−1^. The lowest fruit N content was observed in fruits from “Tyty” F_1_ grown using a NS with a Mo concentration of 0.0 μmol L^−1^.

ANOVA for fruit Mo content showed a significant effect of the interaction genotype × Mo concentration (Table 1). Tomato fruits from “Paride” F_1_ grown using a NS with a Mo concentration of 4.0 μmol L^−1^ had the highest fruit Mo content, followed by those from “Ornela” F_1_ grown with 4.0 μmol Mo L^−1^ which in turn were 83.3, 57.1, 57.1, 83.3, 83.3, and 57.1%, respectively higher than those from “Bybal” F_1_ grown using a NS with 0.5 (standard NS), 2.0 and 4.0 μmol Mo L^−1^, “Tyty” F_1_ grown with 4.0 μmol Mo L^−1^ and “Paride” F_1_ grown with 0.5 (standard NS) and 2.0 μmol Mo L^−1^. Fruits from “Bybal” F_1_ and “Tyty” F_1_ plants grown using a NS with a Mo concentration of 0.0 μmol L^−1^ had the lowest fruit Mo content.

Ignoring of the Mo concentration, genotype did not significantly affect fruit Fe content (Table 2). On the contrary, the Mo concentration significantly influenced the abovementioned parameter. The highest fruit Fe content was recorded in fruits from plants grown with 2.0 μmol Mo L^−1^, followed by those from plants grown using a NS with a Mo concentration of 4.0 μmol Mo L^−1^ which in turn showed a significantly higher value than those from plants grown with 0.0 μmol Mo L^−1^. Nevertheless, tomato fruits from plants grown with 0.5 (standard NS) μmol Mo L^−1^ did not show significant difference neither from fruits from plants grown with 4.0 μmol Mo L^−1^ nor from those from plants grown with 0.0 μmol Mo L^−1^. No significant interaction was found between genotype and Mo concentration in terms of fruit Fe content.

Regardless of the Mo concentration (Table 2), the highest values in terms of fruit Cu content were collected in fruits from “Bybal” F_1_, “Paride” F_1_, and “Ornela” F_1_, whereas, “Tyty” F_1_ revealed the lowest ones. Irrespective of the genotype, fruits from tomato plants grown using a NS with 0.0 and 0.5 (standard NS) μmol Mo L^−1^ revealed the highest values in terms of fruit Cu content followed by those from plants grown with 2.0 μmol Mo L^−1^. The lowest fruit Cu content was observed in tomato fruits from plants grown with 4.0 μmol Mo L^−1^. No significant interaction was found between genotype and Mo concentration in terms of fruit Cu content.

## Discussion

In the soilless cultivation systems, the management of the nutrient solution is a main feature to achieve good yield and fruit quality (Islam et al., 2018). In this article, we studied the effect of diverse Mo concentration in the NSs on yield and fruit nutritional quality of different tomato genotypes. Our results showed that improvements in terms of production, vigor and overall fruit quality can be accomplished using a nutrient solution with a Mo concentration higher than standard dosage (0.5 μmol Mo L^−1^). Molybdenum deficiency negatively affected pollen formation, opening of flowers, capacity of the anther of pollen production, dimension of pollen grains, invertase activity, and pollen germination in maize (Merschner, 2012). Our findings are consistent with those obtained by Longbottom et al. (2010) who, by investigating on the effects of sodium molybdate foliar spray concentration in the vegetative and reproductive structures and on yield components of grapevine cv. Merlot, found that Mo-treatment significantly increased yield of molybdenum deficient vines due to an improved fruit set. Our findings are also consistent with those observed by Kostova and Mehandjiev (2013), who by investigating the influence of fertilization upon the content of molybdenum in tomatoes, found that the yield increased as plant Mo availability increased. However, our results are different from those of Moncada et al. (2018) who reported that an increase of Mo concentration in the nutrient solution had no influence on yield and morphological traits of leafy vegetables grown on floating panels. Our findings are also different from Biacs et al. (1995) and from Vieira et al. (2005) who revealed that no significant change occurred in terms of yield in carrot and bean, respectively as a function of Mo treatment applied to soil or foliage. Our positive response of tomato to Mo could be related either to diverse species studied or to different plant tissue analyses. In accord to the tomato belonging group, the genotypes tested showed a different average fruit weight. However, the different Mo supply levels did not affect the aforementioned parameter. Therefore, our results point out that, although, different species might behave dissimilarly for yield, morphology and vigor traits in function of the Mo concentration in the NS, some biometric traits might not be affected by different Mo supply levels. In our article, quality parameters, especially important for fruit marketability, such as fruit skin color, were evaluated in regard to different genotypes and various Mo concentrations in the NS. According to the scientific literature, tomato fruit color affects the grade and appearance of the end processing products as a result of the presence of different pigments, particularly lycopene (Lancaster et al., 1997; Batu, 2004; Brandt et al., 2006). Tomato fruit is well known as an excellent source of different antioxidants and secondary metabolites such as carotenoids and phenolic compounds (Luthria et al., 2006). Other authors have found that genotype and environmental factors have significant effects on the content of the secondary metabolites in tomato fruits (Dumas et al., 2003; George et al., 2004; Toor et al., 2006). Our results showed that improvement in fruit lycopene and polyphenol contents can be reached using a nutrient solution with a Mo concentration higher than standard dosage (0.5 μmol Mo L^−1^). Considering that Bergmann (1992) and Gupta (1997) found that tomato and cauliflower plants grown at high concentrations of molybdenum showed anthocyanin accumulation in the leaves, we may hypothesized that a higher polyphenol and lycopene accumulation in tomato fruits were due to a greater molybdenum availability.

Ascorbic acid in tomato fruits provides health benefits for humans and also plays an important role in several aspects of plant life (Di Matteo et al., 2010). Cheng (1994) reported that ascorbic acid content increased linearly with Mo application rates for strawberry grown in Mo-deficient, light soil. In our study, with the exception of “Ornela” F_1_, ascorbic acid fruit content in all genotypes decreased as the Mo levels increased. Our different response could be related, as pointed out by Brodrick and Giller (1991), to the fact that relative allocation of Mo to the various plant organs varies considerably not only among plant species, but also among genotypes within a species.

Our results on fruit water content are in agreement with those of Boertje (1969) and with those of Valenciano et al. (2011) and Randal (1969) who found that a Mo implementation caused a significantly increase of dry matter production in chickpea and grain, respectively.

SSC/TA values for all the treatments considered here were higher than 13.0, suggesting that growth in a soilless culture system might impart a sweet and acidic flavor. However, considering that regardless of the genotype, ANOVA did not show significant differences in terms of TA, we can assume that SSC played a principal role on SSC/TA ratio determination.

Kovács et al. (2015) reported that some metals such as Mo, Cu, and Fe are fundamental for the function of the nitrate reductase enzyme and, consequently, play an important role in nitrate reduction. Hence, in our work these metals were also detected. Our results on Mo fruit content are consistent with the findings of Kovács et al. (2015), who observed that the Mo concentration in maize seedlings increased progressively with increasing Mo concentration in the nutrient solution, implying that uptake depends on the amount of Mo supplied to the plants. Furthermore, our outcomes are also in accord with those of Liu et al. (2017), who found that the Mo concentration in strawberry fruits increased gradually with increasing Mo supplied to the plants (from 0.0 to 202.5 g ha^−1^) and with those of Moncada et al. (2018), who revealed that the Mo concentration in lettuce, escarole and curly endive increased with increasing Mo concentration in the nutrient solution of a floating cultivation system. Nitrogen is a basic component of amino acids, proteins, nucleic acids, and quite a lot of other metabolites, which are essential for the growth and development of plants. It is also documented that molybdenum cofactors (Moco) participate in the active site of nitrate reductase, which plays an important role in nitrate assimilation and may improve the utilization rate of the N fertilizer (Schwarz, 2016). Our results on N fruit content are partially consistent with those obtained by Liu et al. (2016, 2017), who revealed that total fruit N content increased with appropriate Mo treatments. On the contrary, Moncada et al. (2018) reported that Mo fertilization resulted to be effective in reducing nitrate content in lettuce at 1.5 μmol L^−1^ and in escarole and curly endive at 3.0 μmol L^−1^. We found a N fruit content increase from 0.0 to 2.0 μmol Mo L^−1^ and a reduction when tomato plants were grown at 4.0 μmol Mo L^−1^. On this respect, we might hypothesize that tomato plants is less sensitive to Mo fertilization than lettuce, escarole, and curly endive.

Our results showed that the Fe fruit concentration increase up to 2.0 μmol Mo L^−^1. According to the literature there is a relationship between Mo and Fe. Our findings are in accord with those of Berry and Reisenauer (1967) who found that the molybdate supply significantly increased the capacity of tomato plants to absorb Fe^2+^. Our findings on fruit Fe concentration are also in agreement with those of Liu et al. (2017) who observed that spraying Mo can enhance Fe concentration in strawberry fruits. On this regard, our hypothesis are in accord with that formulated by Liu et al. (2017), who attributed this response to the fact that the uptake mechanisms for Mo and Fe may affect each other and most Mo enzymes also require Fe-containing redox groups such as Fe-sulfur clusters or hemes.

Kuper et al. (2004) and Llamas et al. (2006) stated that Cu is also associated with Moco biosynthesis. Our outcomes on fruit Cu concentration are in line with those obtained by Kuper et al. (2004), who carried out an *in vitro* study and observed that 1 μmol L^−1^ CuCl_2_ inhibits Moco synthesis, with molybdenum-adenosine monophosphate bound to the cofactors for nitrate reductase and xanthine dehydrogenase, demonstrating competition between Cu and Mo during Moco synthesis. However, our findings are in contrast with those obtained by Liu et al. (2017), who revealed that the Cu concentration in strawberry fruits increased with Mo application rates (from 0.0 to 202.5 g ha^−1^ Mo) and hypothesized that Cu would have a protective role for MPT dithiolate. According to our study, it seems that the relationship between Mo supply and Cu fruit concentration might be attributed to the different genotypes. Moreover, considering the statistical significance of some interactions genotype × Mo concentration in the NS for many of the dependent variables considered, such as total yield, marketable yield, aboveground biomass, a^*^, ascorbic acid, SSC, TA, SSC/TA, N fruit content, and Mo fruit content, our study highlights the different behavior of the tomato genotypes tested when subjected to different levels of Mo concentration in the nutrient solution.

## Conclusion

Molybdenum enrichment of tomato plants significantly affected yield, plant vigor, early flowering, overall fruit quality, and nutraceutical compounds in tomato fruits. Compared with the no-enriched plants (0.0 μmol Mo L^−1^) or with the control (standard NS with 0.5 μmol Mo L^−1^) a Mo fertilization of 2.0 μmol Mo L^−1^ effectively promoted production performance and plant vigor as well as the accumulation of SSC and some antioxidant compounds such as lycopene, polyphenols, and ascorbic acid. Our findings also revealed that the enrichment of Mo in the NS was not detrimental to tomato plants. Lastly, due to the significant interactions (genotype × Mo concentration), this study shows that for each tomato genotype tested there is an optimal Mo concentration in the nutrient solution such that utmost levels of yield and overall fruit quality may be achieved. However, taking all together our results clearly suggest that, a Mo fertilization of 2.0 μmol Mo L^−1^ may successfully improve crop performance and fruit quality of tomato.

## Author Contributions

LS, FD, and GI conceived and designed the research. LS also analyzed the data and wrote the manuscript. AM and ED carried out greenhouse work. CD performed laboratory analytical determination and help with draft the manuscript. All authors read and approved the manuscript.

### Conflict of Interest Statement

The authors declare that the research was conducted in the absence of any commercial or financial relationships that could be construed as a potential conflict of interest.
